# The Gut Microbiota and Oxidative Stress in Autism Spectrum Disorders (ASD)

**DOI:** 10.1155/2020/8396708

**Published:** 2020-10-01

**Authors:** Tingting Hu, Yinmiao Dong, Caixia He, Mingyi Zhao, Qingnan He

**Affiliations:** Department of Pediatrics, The Third Xiangya Hospital, Central South University, Changsha, 410013 Hunan Province, China

## Abstract

Autism spectrum disorders (ASDs) are a kind of neurodevelopmental disorder with rapidly increasing morbidity. In recent years, many studies have proposed a possible link between ASD and multiple environmental as well as genetic risk factors; nevertheless, recent studies have still failed to identify the specific pathogenesis. An analysis of the literature showed that oxidative stress and redox imbalance caused by high levels of reactive oxygen species (ROS) are thought to be integral parts of ASD pathophysiology. On the one hand, this review aims to elucidate the communications between oxidative stress, as a risk factor, and ASD. As such, there is also evidence to suggest that early assessment and treatment of antioxidant status are likely to result in improved long-term prognosis by disturbing oxidative stress in the brain to avoid additional irreversible brain damage. Accordingly, we will also discuss the possibility of novel therapies regarding oxidative stress as a target according to recent literature. On the other hand, this review suggests a definite relationship between ASD and an unbalanced gastrointestinal tract (GIT) microbiota (i.e., GIT dysbiosis). A variety of studies have concluded that the intestinal microbiota influences many aspects of human health, including metabolism, the immune and nervous systems, and the mucosal barrier. Additionally, the oxidative stress and GIT dysfunction in autistic children have both been reported to be related to mitochondrial dysfunction. What is the connection between them? Moreover, specific changes in the GIT microbiota are clearly observed in most autistic children, and the related mechanisms and the connection among ASD, the GIT microbiota, and oxidative stress are also discussed, providing a theory and molecular strategies for clinical practice as well as further studies.

## 1. Introduction

ASD, a loose umbrella term that includes a series of life-long heterogeneous clusters of neurodevelopmental disorders, is characterized by stereotyped behavior and deficits in social communication, interaction, and perception [1]. It is well documented that ASD occurs in all racial, ethnic, and socioeconomic groups. Last updated on July 11, 2016, the US Centers for Disease Control and Prevention (CDC https://www.cdc.gov/) reported an incidence of 1 in 54 children worldwide; in addition, studies in Asia, Europe, and North America have identified individuals with ASD and shown an average prevalence of between 1% and 2% (see Table 1). Additionally, it is conservatively estimated that the prevalence of ASD in China is 1%, among which more than 2 million children aged 0-14 years have the disease [2]. Undoubtedly, ASD, considered a hidden disability, creates an enormous burden on individuals, families, and society [3]. Regrettably, there is no practical and targeted treatment for ASD, which has become a major worldwide health problem [4].

Although the causes of ASD remain unclear, many studies have pointed to the possible link between ASD and multiple environmental as well as genetic risk factors [5]. Of note, much evidence indicates that oxidative stress plays a vital role in the pathophysiology of nervous and mental diseases, particularly in ASD [6–9]. Anecdotal reports have suggested that oxidative stress response is crucially important to the neuroinflammatory response, and in a sense, the neuroinflammatory response has always been regarded as one of the pathogenic factors of ASD [10]. A sibling control study by Shannon et al. suggested that autistic children are more susceptible to oxidative stress on account of an imbalance in glutathione levels inside or outside of cells and a decrease in glutathione (GSH) storage capacity [11].

**Figure 1:**
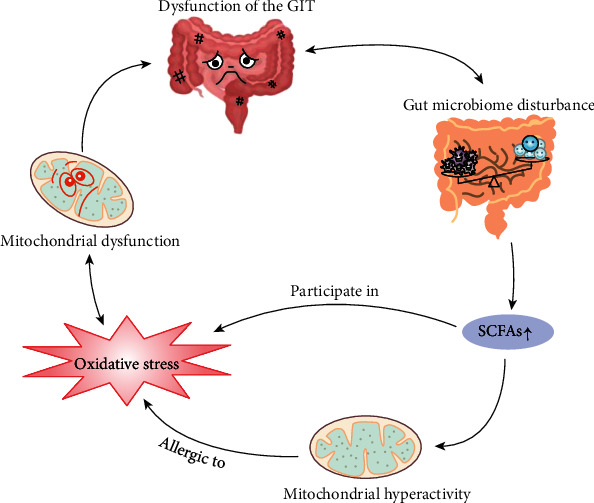
The connections among oxidative stress, mitochondrial dysfunction, and dysfunction of GIT in autistic children. The dysfunction of GIT in autistic children is related to mitochondrial dysfunction, and there is an interaction between oxidative stress and mitochondrial dysfunction. SCFAs, metabolites of the GIT microbiota, not only participate in the reaction process of oxidative stress but also can result in mitochondrial hyperactivity and further make mitochondria allergic to the oxidative stress.

In recent years, as research continues, the research focus of the intestinal microbiota has been rapidly shifting from the abundance and diversity of microbial members to functional aspects. A variety of studies have concluded that the GIT microbiota influences many aspects of human health, including metabolism, the immune and nervous systems, and the mucosal barrier. In addition, the intestinal microbial fermentation of dietary fibres and resistant starch produces short-chain fatty acids (SCFAs), especially butyrate, propionate, and acetate [12]. Notably, propionate can result in GIT metabolic disturbance, reversible behavioral and signalling changes, neuroinflammation, etc. [13]. Therefore, not surprisingly, increasing evidence supports that autistic children are more likely to experience problems related to GIT, including food allergies (FAs), dysbiosis, inflammatory bowel disease (IBD), and indigestion [14, 15]. The GIT dysfunction in autistic children is related to mitochondrial redox imbalance, i.e., mitochondrial dysfunction, and there is an interaction between oxidative stress and mitochondrial dysfunction [16, 17]. Additionally, some connections exist between these factors (see Figure 1). The specific associations are discussed below.

In this review, many aspects of the role of oxidative stress and GIT microbiota in ASD are described. Furthermore, we will discuss, in the context of the most recent literature, the connections among oxidative stress, ASD, and GIT microbiota. Finally, the possibility of oxidative stress and GIT microbiota as therapeutic targets will be discussed, which will provide theoretical basis and novel strategies for clinical practice and future studies.

## 2. Oxidative Stress and ASD

### 2.1. The Definition of Oxidative Stress and the Importance/Role of Reactive Oxygen Species (ROS)

Oxidative stress, considered an out-of-balance state between antioxidants and antioxidants, could lead to the cellular damage caused by reactive ROS or reactive nitrogen species (RNS) [18]. ROS, a kind of signalling molecule, may contribute to cell viability and tissue oxygen metabolism or inhibit the expression of related genes, including antioxidants [19, 20]. When the level of oxidation exceeds the antioxidant defenses, oxidative stress occurs; in addition, different conditions may result in different levels of oxidative stress [21, 22]. On the one hand, when ROS levels are relatively low, cells can appropriately respond to oxidative stress, a condition called “mild oxidative stress” or eustress. On the other hand, the condition that is more reactive to oxidative stress is termed distress, which is reported to be one of the principal causes of neuroinflammation and damage to astrocyte crosstalk resulting in ASD [23, 24].

In light of published references, under normal circumstances, there is a dynamic balance between the production of ROS and the antioxidant capacity of cells [25]. Furthermore, ROS are intermediate products and by-products that are produced in the electron transport in mitochondria [26, 27]. Notably, many enzymes, including superoxide dismutase (SOD) and glutathione peroxidase (GPx), have a high ROS scavenging capacity [8]. However, the specific role of ROS in ASD is still far from understood.

### 2.2. Blood Oxidative Stress Markers in Autistic Children

An increasing amount of evidence has shown that the pathogenesis of ASD is related to the accumulation of oxidized products and the disorder of antioxidant metabolism [28–30]. The role of oxidative stress in the development of ASD has been studied for decades, and a very large number of markers, as revealed by comparing blood levels of oxidative stress markers in autistic patients to those of healthy individuals, have been the focal point of clinical practice (see Table 2) [31–38]. As shown, the blood levels of oxidative stress markers were all decreased or increased to varying degrees in autistic patients compared with healthy controls, indicating that these markers may be used not only to diagnose ASD but also to provide indicators to monitor and guide individual therapy in the clinic [28]. However, the ability to implement these measures as soon as possible is still far from fully accomplished and requires further insightful evidence, clinical reports, and research studies.

### 2.3. Oxidative Stress in the Brain of Autistic Children

**Figure 2:**
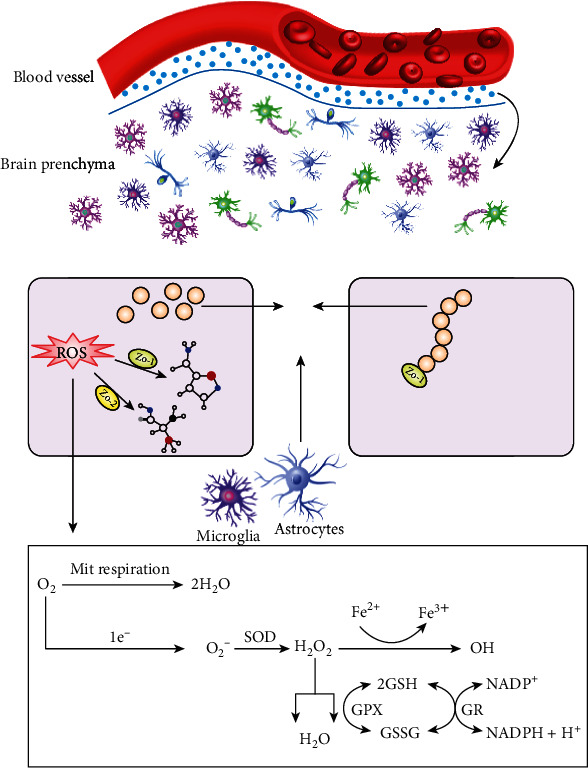
Schematic representation of oxidative stress in the brain.

It is well documented that oxidative stress is a primary potential cause of neuroinflammatory disorders and damage to the blood-brain barrier (BBB), a highly selective boundary that separates circulating blood from the brain and extracellular fluid from the central nervous system (CNS) [39–41]. In healthy individuals, the BBB is formed by tight junctions between tight junction proteins and the endothelial cells of adjacent brain capillaries; then, the tight junction proteins are fixed in the endothelial cells by scaffolding proteins such as ZO-1 and ZO-2 [42]. When oxidative stress occurs in endothelial cells in autistic children, the BBB may be damaged, resulting in varied diffusion and transport [43]. Under normal conditions, ROS from various sources, including mitochondria, microglia, and astrocytes, accumulate. When excessive ROS are not properly scavenged, tight junctions can be altered, leading to further oxidative stress (see Figure 2) [44]. To combat this condition, some mechanisms are required to detoxify or neutralize the oxygen/nitrogen free radicals in the cell (see Figure 2). Devasagayam et al. found that superoxide (O_2_^−^) could be produced as a by-product of normal metabolism; nevertheless, its accumulation may lead to the injury of cell structures and subsequently to oxidative stress [45]. As a result, superoxide enzymes called superoxide dismutase (SODs) immediately convert superoxide to hydrogen peroxide (H_2_O_2_). Moreover, H_2_O_2_ is likely to be toxic to the cells as it can pass through cell membranes, thus damaging DNA. For this reason, scavenging hydrogen peroxide can be a target to disturb oxidative stress and may be a therapeutic intervention for ASD [46, 47]. According to the study by Popa et al., catalase and GPx are considered the most vital enzymes that have the ability to convert H_2_O_2_ to H_2_O. In addition, the tripeptide glutathione, an important antioxidant, plays a vital role in eliminating ROS [46]. Under a reaction catalysed by glutathione peroxidase (GPx), glutathione provides an electron to H_2_O_2_ and is then converted to an oxidized state. By making use of NAD (P) H as the electron donor, glutathione (GSH) could be reproduced again by glutathione reductase. Additionally, glutathione can also obliterate toxic substances from the cells in its role as a cofactor for GSH transferase [46, 48]. In general, the brain is very sensitive to the accumulation of radicals such as ROS on account of the relatively weak protective mechanisms [49, 50].

## 3. Gastrointestinal Tract (GIT) Microbiota and ASD

### 3.1. GIT Microbiota and Factors That Affect the GIT Microbiota

In recent years, as research continues, the research focus of the GIT microbiota has been rapidly shifting from the abundance and diversity of the microbial members to functional aspects. A variety of studies have concluded that the intestinal microbiota influences many aspects of human health, including metabolism, the immune and nervous systems, and the mucosal barrier. The intestinal microbial fermentation of dietary fibres and resistant starch produces short-chain fatty acids (SCFAs), especially butyrate, propionate, and acetate [12]. Butyrate is a key energy substrate for colonocytes [51] and can drive the energy metabolism of colonocytes towards *β*-oxidation by stimulating PPAR-*γ* signalling and limiting the luminal bioavailability of oxygen, maintaining homeostasis, and preventing gut microbiota dysbiosis [52]. Propionate regulates satiety signalling and gluconeogenesis in the liver, protecting the host from diet-induced obesity and associated glucose intolerance [53]. Other metabolites are produced by intestinal microbiota, and additional clinical evidence is needed to fully investigate their functions in physiology and pathophysiology. Examples include indole propionic acid, which seems to improve mucosal integrity in the gut [54], and ethylphenyl sulfate, which is connected to the exacerbation of autistic behaviour in a mouse model [55]. In addition, the intestinal microbiota has also been shown to have a positive impact on glycaemic control [56], energy homeostasis [57], lipid metabolism [58], and protein metabolism [59]. For the immune and nervous systems, the intestinal microbiota modulates the maturation and function of tissue-resident immune cells in the central nervous system (CNS), including microglia and astrocytes; they also influence the activation of peripheral immune cells, which regulate responses to neuroinflammation, brain injury, autoimmunity, and neurogenesis [60]. In addition, the healthy gut microbiota not only plays a dominant role in reinforcing the immunologic barrier [61] but also maintains the structural integrity of the intestinal mucosal barrier [62]. In summary, the GIT microbiota plays vital roles in human physiology and pathology.

As the GIT microbiota is a popular area in therapeutic research, several factors that contribute to the shaping of the normal GIT microbiota have been demonstrated. The mode of delivery (vaginal or caesarean), the local environment (i.e., mother and hospital), and the type of feeding (breast or formula) are significant factors that impact GIT microbiota composition during the neonatal period, resulting in changes that persist until infancy [63–65]. Age also plays an important role in shaping the gut microbiota. It is widely believed that mammals are first exposed to the microbiota in utero and that the microbiota expands rapidly after birth [66]. Studies have also shown that young children and adolescents have a significantly higher abundance of *Clostridium* and *Bifidobacterium* than adults [67, 68]. Diet can also flexibly modulate gut microbiota composition. Just a four-day administration of entirely animal-based or plant-based diet is sufficient to lead to significant shifts in the human gut microbiota [69]. In general, the intake of a diet rich in fruits, vegetables, and fibres is associated with a higher richness and diversity of the gut microbiota [70]. While antibiotics are usually used for saving lives in the fight against infectious disease, many studies have shown their effect on gut bacterial ecology in recent years. The major changes in the GIT microbiota in response to antibiotics include diminished taxonomic diversity and persistence of the changes in a substantial proportion of individuals [71, 72]. In addition to the factors mentioned above, the gut microbiota configuration of individuals is affected by many other factors, including the genotype of the host, ethnicity, and sex [73–75].

### 3.2. Relationship between the GIT Microbiota and ASD

The study by Wang et al. showed that GIT symptoms, such as constipation (20%) and diarrhoea (19%), are more common in autistic children than in healthy children [76], similar to the results of meta-analyses by Coury et al. and McElhanon et al. [77, 78]. In addition, Buie et al. found that autistic children with GIT symptoms may display more apparent behavioral manifestations including anxiety, automatization, and aggression [79]. An increasing amount of evidence has shown that the GIT microbiota is directly or indirectly related to the symptoms of autistic children, mostly by affecting the mucosal immune system and human metabolism [3, 80]. According to a study on laboratory animals published online by the journal *Cell*, GIT barrier defects and GIT microbial disorder occur in mouse models of ASD; additionally, the abundance of *Porphyromonadaceae*, *Prevotellaceae*, *Bacteroidales*, and *Lachnospiraceae* in the offspring of mothers with maternal immune activation (MIA) was greater than that of the controls. Notably, the abundance of *Ruminococcaceae*, *Erysipelotrichaceae*, and *Alcaligenaceae* was greater in the controls [81]. The evidence shown by Finegold et al. demonstrates that the GIT microbiota of autistic children has a greater abundance of *Lactobacillus*, *Clostridium*, *Bacteroidetes Desulfovibrio*, *Caloramator*, and *Sarcina* as well as lower levels of *Bifidobacterium* and *Firmicutes* than the GIT microbiota of nonautistic children [82]. Furthermore, autistic children with GIT symptoms show lower levels of *Prevotella*, *Coprococcus*, and *Veillonellaceae* than autistic children without the abovementioned symptoms [83]. As stated, numerous studies have demonstrated the changes in the GIT microbiota of autistic children, but the relationship between GIT microbiota and ASD is relatively unexplored.

**Figure 3:**
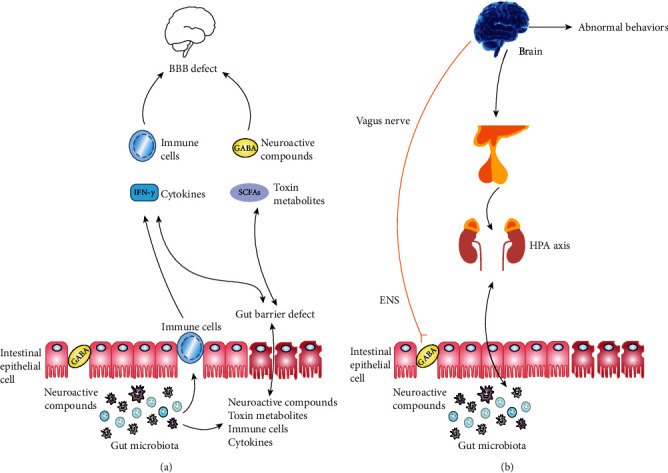
Relationships between the GIT microbiota and ASD (the microbiota-gut-brain-axis). Note: BBB: blood-brain barrier; ENS: enteric nervous system; GABA: *γ*-aminobutyric acid; HPA: hypothalamic-pituitary-adrenal; SCFAs: short-chain fatty acids.

In recent years, as study continues, the microbiota-gut-brain-axis has been considered a bidirectional physiological communication between the brain, the GIT microbiota, and the GIT; not surprisingly, accumulating evidence demonstrates that this axis is related to the aetiology and pathogenesis of ASD [84–86]. As shown in Figures 3(a) and 3(b), the metabolites produced by the GIT microbiota, especially SCFAs, can pass through enterocytes (ECs) to have an impact on the function of the brain. In addition, some kinds of GIT microbiota can generate neuroactive substances such as 5-HT and GABA, which can also pass through the EC, affect the function of the brain, and further lead to unexpected behaviors [87]. On the one hand, neuroactive substances, some GIT microbiota, and metabolic products could activate neurons in the brain and act on the function of the brain via vagus nerves. On the other hand, among the abovementioned substances, the neuroactive substances that directly affect the hypothalamic-pituitary-adrenal (HPA) axis can ultimately increase circulating levels of cortisol. Additionally, some GIT microbiota and metabolic products could also activate and induce GIT-immunizing cells to liberate cytokines (CKs) to play a corresponding role in the body's circulation [88, 89].

An increasing amount of evidence suggests that modulation of the GIT microbiota is a potential therapy in autistic children by elucidating the relationship between the GIT microbiota and ASD, including faecal microbiota transplantation (FMT), probiotics, and dietotherapy. The literature on ASD treatments linked to the GIT microbiota in the past five years is summarized and analysed in Table 3 [90–95]. First, probiotics are considered to prevent intestinal diseases through functions such as regulating and controlling the blood-brain barrier (BBB) and gap-associated proteins [96]. The recently developed approach FMT is an intervention in which the faecal microorganisms from healthy persons are delivered to patients with bad GIT microbiota [97]. However, although many scholars and experts have speculated about the safety of FMT, but no final conclusions have been reached on this matter. Of course, the other abovementioned therapies also have their own limitations (see Table 3). In my opinion, additional well-designed studies with a larger sample size are needed to offer further evidence supporting the feasibility of these treatments.

## 4. The Relationship among GIT Microbiota, Oxidative Stress, and ASD

**Figure 4:**
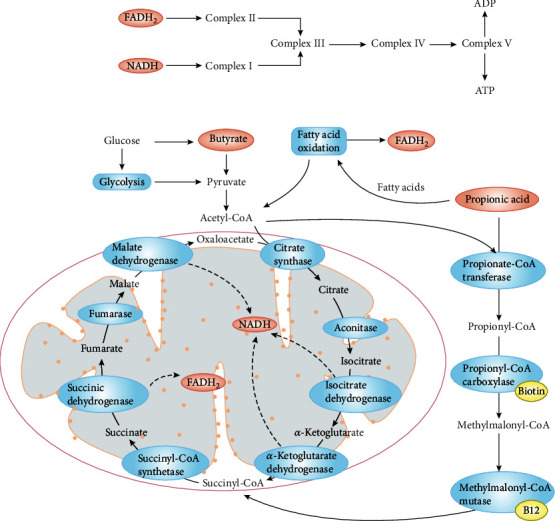
Mitochondrial pathways involved in SCFAs as substrates. There are two different starting points in the electron cycle chain, i.e., Complex I and Complex II, which have their exclusive fuel sources. Notedly, Complexes III, IV, and IV are all involved in the abovementioned reactions; furthermore, butyrate and propionic acid enter into mitochondria to participate in related reaction via two crossed and overlapped pathways. Butyrate which resembles the glucose commonly enters into the citric acid (TCA) cycle via Acetyl-CoA, a key reaction substance. The TCA cycle mainly generates a kind of substrate of Complex I called Nicotinamide adenine dinucleotide (NADH). FADH2, as the substrate of Complex II, can be massively produced in two varied metabolic pathways which propionic acid participates in. Equally, propionic acid can produce some substrates of oxidative stress such as SCFAs et al. to be involved in related responses.

As discussed above, the relationship between GIT symptoms and ASD via mitochondrial dysfunction is quite convincing and worthy of study, as it is well documented that ASD is linked to GIT symptoms and mitochondrial dysfunction; additionally, the latter two are strongly related [98]. According to authoritative reports, the GIT, as an available site, can induce the production of SCFAs such as PPA and BUT [99]. Additionally, GIT dysfunction is observed in autistic children, such as the increased abundance of *Clostridia* spp., which produce PPA and BUT. There is no doubt that both PPA and BUT can regulate metabolism, including acting as mitochondrial fuels [100], despite entering into the mitochondrial energy pathways at slightly different sites (see Figure 4) [101]. In addition, SCFAs impair the physiological function of cells, resulting in further GIT symptoms related to ASD, including nonspecific inflammation [79, 93, 102].

To verify whether a connection among the GIT microbiota, oxidative stress, and ASD truly exists, Shannon et al. performed a blinded case-control study and unsurprisingly found differences in the function of mitochondria during several enzymatic reactions in autistic children compared to control children, indicating that differences actually exist in mitochondria rather than in certain enzymes. These researchers also found that the mitochondrial physiology of the GIT in autistic children differs from that in healthy children; notably, the discrepancies are particularly prominent in the caecum [17]. We speculate that autistic children might have different mitochondrial parameters, particularly in the caecum.

Mitochondria are characterized by energy generation; in addition, approximately 5% of autistic children exhibited impaired energy generation to make energy, and more than 30% of autistic children exhibit elevated biological markers. Therefore, we hypothesized that autistic children may have nontraditional mitochondrial diseases [103]. Similarly, a sibling control study found that lymphoblastoid cell lines (LCLs) from autistic children are active in the mitochondrial respiration process, which further leads to a greater sensitivity to oxidative stress [11]. Indeed, PPA and BUT are also abundant in the caecum, which indicates a role for the GIT microbiota in mitochondrial dysfunction in autistic children [104]. In general, the abovementioned correlated responses could result in a clearer understanding of the pathological relationship linked to GIT dysfunction and oxidative stress in ASD, which provides a theoretical basis as well as molecular strategies for new treatment paradigms.

## 5. Novel Therapeutic Approaches for Oxidative Stress in ASD

Based on the relationship discussed above, we will next discuss, in the context of the most recent literature, the possibility of novel therapies regarding oxidative stress as a target (Table 4) [105–108]. It is well documented that COX-2, as a vital enzyme that is overexpressed in tissues under oxidative stress, affects the metabolism of polyunsaturated acid (PUFA). *ω*-3 is also considered to be connected with the high expression of COX-2. Leukotrienes are reported to decrease the expression or activity of an iron-containing dioxygenase named 5-LOX, ameliorating neuroinflammation, restoring normal synaptic plasticity, and improving learning ability and memory [11]. Additionally, docosahexaenoic acid (DHA) and *ω*-3 are both needed for the appropriate growth and development of the brain, for proper synapse formation and to improve cognitive function [104, 105]. Notably, the use of vitamin B12 as a novel therapeutic approach that has recently aroused public attention has been shown to be used for the treatment of ASD; however, its efficacy is unclear [105].

## 6. Concluding Remarks

Many reports have highlighted the relationship among the GIT microbiota, oxidative stress, and ASD. A number of studies emphasize the important role of oxidative stress, which is connected to mitochondrial dysfunction, GIT microbiota disturbance, and thus the production of various metabolites. This review has summarized the latest research on the related mechanisms, but most of the authors have only concentrated on individual pathogenic mechanisms or metabolites; thus, there is an urgent need for studies considering broader biochemical pathways. Additionally, among all the novel ASD therapies mentioned above, the potential usefulness of most have been investigated. Although the results are promising, these therapies are still thought to have limitations and lack safety testing. In general, the author thinks that additional well-designed studies with a larger sample size are needed to offer further evidence supporting the feasibility of these treatments. In addition, the appropriate dose and timing of therapy also require further study.

## Figures and Tables

**Table 1:** Summary of the Prevalence of ASD in different areas.

Country	Age range studied	Number of children in population	Criteria used	Methodology used	ASD prevalence (CI)
USA	8	346,978	DSM-IV	Case enumeration and record review	14.6 (8.2-24.6)
Faroe Islands	7 to 16	7122	DSM-IV, ICD-10	Screening and direct exam	5.6
Denmark	N/A	404,816	DSM-IV	Case enumeration	6.9 (6.5-7.2)
Oman	0 to 14	798,913	DSM-IV	Case enumeration	0.1 (0.1-0.2)
Taiwan	0 to 18	372,642	ICD-9	Case enumeration	2.9
South Korea	7 to 12	55,266	DSM-IV	Case enumeration from survey and direct exam	26.4 (19.1-33.7)
Western Australia	N/A	152,060	DSM-IV	Case enumeration	5.1 (4.7-5.5)

Note: data are from the US Center for Disease Control and Prevention (CDC https://www.cdc.gov/). N/A: not applicable, i.e., the lack of data in a form or table.

**Table 2:** Blood levels of oxidative stress markers in autistic patients.

Marker/specimen	Units	Values ASD		*p* value	Reference
		Autistic children	Controls		
Lipid hydroperoxide (LOOH) in the temporal cortex	mmol/mg protein	About 21^#^	About 15^#^	<0.05	[31]
Plasma malondialdehyde (MDA)	nmol/mL (mean ± SD)	4.16 ± 1.67	1.49 ± 0.58	<0.001	[32]
Serum malondialdehyde (MDA)	nmol/mL (mean ± SD)	8.6 ± 0.5	1.76 ± 0.33	≤0.001	[33]
RBC thiobarbituric acid reactive substances (TBARS)	mmol/g Hb (mean ± SD)	0.032 ± 0.0077	0.015 ± 0.0033	<0.001	[32]
Plasma protein carbonyl	nmol/mL (mean ± SD)	4.202 ± 0.3912	2.256 ± 0.148	<0.0001	[34]
Serum 8OHdG	ng/mL (mean ± SD)	13.134 ± 1.33	1.46 ± 0.326	≤0.001	[33]
Plasma glutathione peroxidase (GPx)	U/L (mean ± SD)	40.9 ± 11.3	24.2	<0.0001	[35]
Serum catalase (CAT)	UAE/L (mean ± SD)	2.836 ± 0.479	0.689 ± 0.157	≤0.001	[33]
RBC catalase (CAT)	k/g Hb (mean ± SD)	209.31 ± 61.92	515.77 ± 127.9	<0.001	[32]
RBC superoxide dismutase (SOD)	U/g Hb (mean ± SD)	2123.59 ± 543.53	971.31 ± 239.14	<0.001	[32]
Plasma reduced glutathione (GSH)	*μ*mol/L (mean ± SD)	3.1 ± 0.53	4.2 ± 0.72	<0.0001	[36]
Plasma glutathione (GSH)	*μ*mol/L (mean ± SD)	3.14 ± 0.56	4.2 ± 0.72	<0.0001	[37]
Plasma oxidized glutathione (GSSG)	nmol/L (mean ± SD)	0.48 ± 0.16	0.35 ± 0.05	<0.001	[37]
MT-1A expression in blood	N/A	Higher (no data available)	N/A	≤0.001	[38]

Note: the data are from references 31–38. RBC: C red blood cell; SD: standard deviation. ^#^Values were estimated from the figure. N/A: not applicable, i.e., the lack of data in a form or table.

**Table 3:** Literatures on the treatments of ASD linked to GIT microbiota.

Model	Behavior tests	Treatments	Dosages	Time	Effects	Limitations	Year	References
10 autistic children, 9 nonautistic siblings, 10 control	CARS and ADI	Probiotic including *Lactobacillus*, *Bifidobacteria* and *Streptococci*	One pill three times a day	4 months	Increased abundance of the *Bacteroidetes/Firmicutes*, normalized the amount of Bifidobacterium and Lactobacillus can decrease the TNF*α* levels in the autistic children.	No follow-up was performed after treatment	2015	[90]
A 12-years-old boy with ASD, severe cognitive disability	ADOS-2	Probiotic (*lyophilized bifidobacteria*, *lactobacilli*, and *Streptococci*)	9 − 20 × 10^10^	4 weeks	Reduced GIT symptoms and improved in dominating autistic symptoms	More well-designed studies with a larger sample size are needed to offer more proofs supporting the feasibility of it.	2016	[91]
3 autistic child, 3 nonautistic children	N/A	Prebiotic: galactooligosaccharide and B-GOS	2 g	Everyday	Increased abundance of *Bifidobacterium spp*, acetate, and butyrate	It is in an in vitro gut model system	2017	[92]
18 autistic children	PGI-III and CARS	Microbiota transfer therapy (MTT)	Vancomycin (40 mg/kg per day)	2 weeks	Improved both GIT and ASD-related symptoms; normalized the microbiota of autistic children	No placebo controlled, blinded or randomized	2017	[93]
ASD animal model	Self-grooming evaluation, three chambers social test	Ketogenic diet	N/A	2 weeks	Prevention of autism symptoms	The ketosis and glucose levels were not measured	2016	[94]
C57BL/6 and BTBR mice	Three-chamber sociability test et al.	Ketogenic diet	N/A	2 weeks	Decreased all host bacterial abundance in cecal and fecal matter	N/A	2016	[95]

Note: N/A: not applicable, i.e., the lack of data in a form or table. The data are from the references 90–95. CARS: The Childhood Autism Rating Scale; ADI: Autism Diagnostic Interview; ADOS-2: Autism Diagnostic Observation Schedule-2; GI-III: The Parent Global Impressions III.

**Table 4:** The novel therapies of ASD regarding the oxidative stress as a target.

Drugs	Pesticide effect	References
Leukotrienes	Inhibition of the expression or activity of 5-LOX; ameliorate neuroinflammation; restore normal synaptic plasticity; Improve learning and memory function in depressed rats	[105]
Docosahexaenoic acid (DHA)	Be good for the growth and development of the brain and effective at improving cognitive function	[106]
*ω*-3	Be needed for the appropriate growth and development of the brain, proper synapse formation, and to improve cognitive function	[107]
Vit. B12	Normalization of the Hcy level and amelioration of impaired lipid metabolism	[108]

Note: the information are from references 105–108.
